# A Qualitative Exploration of Parental Views When Comparing Individual to Group Sports in Children with Autism Spectrum Disorder—A Pilot Study

**DOI:** 10.3390/ijerph19116906

**Published:** 2022-06-05

**Authors:** Daniel Vella Fondacaro, Francesca Vella Fondacaro, Nigel Camilleri

**Affiliations:** 1 Mental Health Services, ATD 9033 Attard, Malta; nigel.a.camilleri@gov.mt; 2 Faculty of Medicine and Surgery, University of Malta, MSD 2080 Msida, Malta; 3 Mater Dei Hospital, MSD 2090 Msida, Malta; francesca.curmi@gov.mt

**Keywords:** children, autism, sports, physical activity, parents, screen time, qualitative

## Abstract

(1) Background: Physical activity is important for children with autism spectrum disorder. This study aimed to analyse autistic children’s and their parents’ preferences between group and individual physical activity, while exploring potential social barriers that they might encounter. (2) Methods: Retrospective analysis identified 701 new referrals received by the Maltese national child and adolescent mental health service, between 2016 and 2017. Of them, 24 received a sole diagnosis of autism and 10 were chosen via purposive sampling. A semi-structured interview guide was created, including readability testing, translation/back-translation, inter-rater agreements, and focus group testing. Parents were informed, consented, interviewed and thematic analysis carried out. Further quantitative data were tabled accordingly. (3) Results: Only one child met World Health Organisation recommendations for physical activity. More children preferred individual sports while parents described more benefits with group sports. Parents’ perceived benefits with group sports included better socialization, while improved levels of self-esteem and coping with anxiety were highlighted benefits for individual sports. Parents felt misunderstood, burnt out, and described a lack of autism-friendly sports facilities, including geographical disproportionation of adequate facilities on the island. Too much screen time was a major parental concern. (4) Conclusion: Recommendations aim to develop sport therapy systems and well-resourced services in Malta. Staff training is recommended to improve service quality.

## 1. Introduction

It is widely accepted and consistently evidenced within the published literature that physical activity (PA) has a medium-to-large significant positive psychosocial impact on young people’s (YP’s) mental health (ES = 0.748 for resilience; ES = 0.405 for positive mental health; ES = 0.877 for well-being) [1], especially on their self-esteem and social interaction [2]. The World Health Organisation (WHO) recommended that YP from ages 5 to 17 years should carry out at least sixty minutes of moderate-to-vigorous-intensity PA daily [3]. Despite widespread promotion of an active lifestyle, YP have become less physically active in recent years [4], but a wider range of exercises and sports may encourage participation [5]. Younger children carry out more PA when supported by their parents, while older teenagers choose to be physically active due to body image and to form social networks [6].

PA in teams helps YP with autism spectrum disorder (ASD) to improve their social communication and interaction skills by providing opportunities to socialise and meet new people in group activities [2,7]. PA programmes with individual activities were also noted to have several advantages in YP with ASD as it can be adjusted to the individual’s specific needs [8]. Choosing between group and individual PA often becomes a topic of debate amongst parents in child and adolescent mental health services. YP with ASD enjoy group activities less than typically developing peers [9], and only tend to engage in more peer interaction as they grow older [10]. YP might engage better in sports if they choose the type of sports based on preference, rather than social norm or parental opinion [11]. Increasing the PA levels in these YP might help improve ASD symptomatology, which is often associated with parental stress and decreased parental efficacy [12]. The two-part research question for this study was: (1) Do YP with ASD prefer group or individual PA? (2) What difficulties do parents of YP with ASD encounter when trying to help their children carry out their preferred PA? Therefore, this study aimed to qualitatively analyse parental views about individual and group PA in YP with ASD, including the difficulties they encounter.

## 2. Materials and Methods

### 2.1. Participants

New case referrals of YP (5 to 17 years of age) who were diagnosed with ASD via a multimodal assessment (as described by the NICE guidelines, UK: CG128, 28 September 2011) and who attended the National Child and Young People’s Services (CYPS) department, Malta, between 2016 and 2017, were recruited to this study. These YP were identified through a patient electronic database (n = 701) and were selected for this study via purposive and random sampling (Phase 1; Table 1). YP with other comorbid mental disorders other than ASD were excluded from the study. CYPS is the main community-based clinic for the general child and adolescent national mental health service in Malta.

A retrospective case note review of the new case referrals identified 83 YP with a diagnosis of ASD. Twenty-four of these had ASD as their only psychiatric diagnosis (n = 24) and were thus chosen by purposive sampling. Of these, the initial sample for interviewing (n = 10; Table 1) was chosen by random sampling (by lot), which abided by thematic analysis guidelines [13]. The sample of ten people, however, was not fixed in advance. At the end of the tenth interview, it was noted that themes and subthemes were being replicated, while no new contributions to the analysis were being observed. This suggested a level of completeness or category saturation [14], and data generation was concluded.

YP were divided into groups according to their level of severity as per the Diagnostic and Statistical Manual of Mental Disorders, 5th Edition [15]. The levels of severity ranged from Level 1 (‘requiring support’) to Level 3 (‘requiring very substantial support’) and YP were allocated to these groups based on ASD multimodal assessments obtained from the case notes, such as the Autism Diagnostic Observation Schedule (ADOS) and clinical observations. The parents of the YP in the final sample were recruited for participation (P1 to P10). All data were stored anonymised in a password-protected spreadsheet.

All participants were of Caucasian ethnicity and were all Maltese, residing in Malta during the period of research. Six of the participants were female and four were male. Participants had a diverse educational and employment background. The ages of the participants’ children were between five and ten years (10 years, 5 years, 8 years, 5 years, 8 years, 5 years, 5 years, 5 years, 9 years, and 8 years).

### 2.2. Data Collection

#### 2.2.1. Semi-Structured Interview Guide

The interview guide was created by the researcher for the purpose of this study. A semi-structured interview approach was chosen due to its flexibility, allowing the participants to elaborate on various topics which were not predetermined by the researcher and exploring further sensitive issues [16]. Readability testing was carried out and the guide was found to be age appropriate. Translation and back-translation from English to Maltese was carried out by a University of Malta graduate in the Maltese language. The guide was piloted in a focus group and amended accordingly following suggestions brought out by the group and discussion with the department’s clinical lead.

The guide included 11 questions which provided a platform for an open-ended discussion. The guide focused on the type and frequency of PA carried out by the YP and their parents, including the importance they gave to PA in their lives. It also explored the parents’ and YP’s preferences between group and individual PA. The guide focused on barriers and opportunities to perform PA, and the number of hours per week that YP spent using technological devices (screen time) such as television, video games, or computer/tablet. The Godin Leisure-Time Exercise Questionnaire (GLTEQ) was filled in during each interview. The GLTEQ is a validated questionnaire used to monitor for PA [17] and calculates a score by multiplying the hours of strenuous exercise by nine, the hours of moderate exercise by five and the hours of mild exercise by three. The parents filled in two sets of GLTEQ, one for themselves and one on behalf of their children.

#### 2.2.2. Focus Group

A focus group discussion was carried out prior to the interviews. It was facilitated by two research assistants (foundation year doctors during their psychiatry rotation). These were selected to reduce an observation bias from the researcher. The group consisted of three parents of YP with ASD who were randomly chosen from the list of CYPS referrals. The interview guide was discussed and feedback was gathered. This ensured that the questions being asked were found to be acceptable in terms of content, clarity, relevance, and completeness. Following this, an inter-rater exercise was done where two research assistants carried out separate interviews on the same individuals, followed by a consensual discussion between the research assistants together with the main researchers, finalizing the interview guide.

#### 2.2.3. Interviewing Process

Six mothers and four fathers of YP (n = 10) were interviewed. Interviews were carried out individually at the CYPS department and the Psychiatric Unit at Mater Dei Hospital (Msida, Malta) in May 2018 and lasted approximately one hour. Both verbal and written consent were obtained, and all interviews were digitally recorded and transcribed verbatim. Participation was entirely voluntary; every participant was allowed to decline at any point within the interview without giving a reason. No incentives were promised.

### 2.3. Data Analysis

Inductive thematic analysis was used as the coding was not done in line with any pre-existing theory [18]. In addition to thematic analysis, and despite the small sample size, simple descriptive statistics (without significance testing) were carried out to analyse the rest of the data. This included the frequency of PA, the weekly hours of screen time (hours/week) and the number of parents/YP preferring group PA or individual PA.

### 2.4. Ethical Information

The application for an ethical opinion was submitted on 24 December 2017. A favourable ethical opinion (ref: HEC 14/17) was received from the Health Ethics Committee (HEC), Ministry of Health—Government of Malta (date: 29 January 2018). Research governance approval was granted by the Chairman of the Department of Psychiatry, Malta. Both verbal and written consent were obtained from the participants.

## 3. Results

### 3.1. Thematic Analysis Results

Ten interviews were successfully carried out with no dropouts. Thematic analysis generated 169 initial codes. These codes were reviewed and re-grouped into 21 initial themes, which yielded seven final themes (Table 2).

#### 3.1.1. Group Sports, Social Interaction, and Communication

Almost all parents (n = 9) described their children’s social interaction and communication as passive and repentant, especially when meeting new people (n = 8): *“So, I think, erm, what he suffers most is to make friends… He finds it very difficult to write. He finds it difficult to, erm, communicate with other children because he does not talk yet.”* (P2).

Several parents (n = 4) highlighted their children’s lack of confidence: “Maybe sometimes someone tells him, ‘Come play with me!’. And he goes, but only for a while. But they must tell him. He’s not going to go himself…” (P4).

Some parents (n = 3) preferred group sports to individual sports for their children; others (n = 6) explained that both group and individual sports are important in YP with ASD, while only one parent preferred individual to group sports for her child. Some parents (n = 2) believed that group sports help their children’s social interaction by exposing them to other children with different characteristics: *“It helps you integrate more as a group, eh. In today’s society, you must function as a group…”* (P4). Other parents (n = 6) felt that group sports increase motivation and social drive: “*I think it’s better if he’s in a group. Because they will have that small push… It’s like, in a group you’re not alone…”* (P10).

#### 3.1.2. Individual Sports, Self-Esteem, and Venting One’s Frustration

Parental interviews reported that four YP preferred individual sports to group sports, four YP preferred both group and individual sports, while two YP preferred group to individual sports. Despite the general parental preference towards group sports, some parents explained that individual sports might also have some benefits, including self-esteem: *“…then maybe because it’s individual sports, it muffles his shyness and boosts his self-esteem basically.”* (P1), and confidence: “*So individual sports is important because it gives you confidence”* (P7). Some parents (n = 3) also felt that individual sports may serve to tackle hyperactivity and help cope with anxiety in YP with ASD: *“The more he runs, the more he takes out his frustration.”* (P1).

Parents (n = 3) also mentioned the importance of giving children an active role when choosing their sport or the type of PA as it improves the child’s willingness to attend: “*…if he does not know how to play football, it’s useless putting him in a football match alongside other children. He does not know how to play, so he won’t integrate or else runs out.”* (P2).

#### 3.1.3. Lack of Public Awareness, Opportunities, and Resources

Most parents (n = 6) spoke about the lack of public awareness and the negative effects of stigma on ASD: *“People, eh, they make it even more difficult… Because there is no awareness. I was one of them…”* (P10). Others (n = 2) felt that awareness is slowly improving, especially between YP themselves: “*Back in the days, I’m 35, we never used to see what we see today. We used to see someone like this and laugh at him.”* (P7)

Parents (n = 7) spoke about the lack of opportunities and ASD-friendly resources and facilities: *“… whether they are teachers or coaches, whatever… they are not qualified for the job, with autism.*” (P7). This included the lack of human resources and one-to-one attention: “*…for example, my nephews and nieces go to a lot of sports and drama. But I can’t register my son because he needs someone one-to-one.”* (P2).

Most parents (n = 6) spoke in favour of grouping YP with ASD in special groups to facilitate participation. Others (n = 2) preferred mixed ability groups as it helps YP with ASD to learn socially appropriate behaviours from other neurotypically developing children: *“I think he needs to adapt to the sport. He has to be able to cope. It doesn’t matter if he doesn’t manage immediately…”* (P3).

#### 3.1.4. Lack of Motivation in Sports

Parents (n = 3) spoke about the lack of motivation that their children experience when carrying out sports and how this hinders future participation: “*He does karate, and he started getting tired. Yes, yes. He tries, let me say that, but, erm, it’s like when he sees difficulty, he stops. He gives up very easily.”* (P3). They also spoke about how motivation is bolstered by peer-to-peer encouragement: *‘Because if they have that small push, in a group not alone, you’ll make a bigger effort.’* (P 10).

Parents (n = 4) associated the child’s lack of motivation with the home environment. They explained that their children participated more in PA sessions when organised at school rather than at home: “*But the learning support assistant does a lot more with them than us. She tells us stuff that we can never imagine him doing at home. He does them at school…”* (P10).

#### 3.1.5. Physical and Mental Burden on Relatives

Parents (n = 5) felt that parenting a child with ASD contributes to more physical and mental stress: “*I have fibromyalgia as well! So, I have my own problems. It might be that the fibromyalgia came out because of these children*.” (P6). Parents also felt that they had to adapt their personal lifestyle to better attend to their children’s needs: “*I don’t have time for myself or to do any sports. But what I do with him is, we go run… But if we go run, and if he is going to run, then I have to run with him*.” (P2); “*I have a spinal condition, and I’m going to physiotherapy, and I’m not finding the time to do them. All I need is twenty minutes a day, and I’m not finding the time…*” (P7).

Some parents (n = 3) reported that sports facilities are located too far away from their residential home. They suggested a more even geographical distribution, making it more convenient for everyone to attend: “*Not, for example, in Valletta… but in the South. Because like I’m telling you, to go to the North with two children, it is a bit difficult for me.”* (P8).

#### 3.1.6. General Improvement in Mental and Physical Health

Despite the reported impact that their child’s diagnosis had on the family’s lifestyle, parents reported that symptoms gradually improved with age: “*For example, he used to stay rotating the wheels of his toy cars I used to give him, all the time. Now he reduced these habits a lot, thank God.”* (P10).

Parents (n = 6) felt that ASD symptoms improved with PA. Some explained that this might be due to the calming effect of PA and the promotion of a healthy lifestyle: “*It’s good for physical health, and mentally as well I think, because I think sports, at the same time, relaxes you as well. It’s like you escape from life and the daily stresses.”* (P4).

#### 3.1.7. Technology and Screen Time

One of the issues which greatly concerned parents was the excessive number of hours which their children spent using technological devices. They felt that excessive screen time had a negative effect on their children’s PA patterns and school performance: “*When he comes home, he does not move from his chair between three and eleven. Playing PlayStation. It’s like I have a man who’s hooked on wine, and I cannot take it away from his hands.”* (P6).

However, two parents spoke about the role technological devices have on their children’s education. They explained that technological devices may help with visual learning: “*They watch YouTube. So, they rarely watch a film. But still, we always try to show her educational stuff as much as possible.”* (P7).

### 3.2. Quantitative Results

#### 3.2.1. Physical Activity

Only three parents reported that their child does extracurricular PA or sports which follow a weekly schedule. All parents agreed that PA is important for YP with ASD (nine ‘strongly agreed’ and one ‘agreed’). Parents generally preferred group sports while YP generally preferred individual sports. All ten participants were asked to fill in a GLTEQ for themselves and one for their children. The mean GLTEQ scores for both parents and YP were highest in the level 3 ASD severity group (42.75 and 54.75, respectively) and lower in the Level 1 and Level 2 ASD severity groups (Table 3).

#### 3.2.2. Screen Time

Parents generally expressed their concern with daily long hours of screen time. Some (n = 2) felt that this was used as a mechanism for coping with stress. Reported mean screen time (hours/week) was highest in YP with Level 1 ASD severity (48 h/week) and lowest in YP with Level 3 ASD severity (15.125 h/week), as shown in Table 4. It was observed that YP who were reported by their parents to engage in lower levels of physical activity were also reported to spend more hours of weekly screen time.

## 4. Discussion

To the authors’ knowledge, this is the first study of its kind in Malta and it can serve as a platform for further research as well as service development of ‘autism friendly’ sports facilities. One of the key findings from this study was an overall lower level of PA in YP with ASD, when compared with WHO recommendations [3]. Another key finding was the lack of engagement (n = 2) in group PA by YP with ASD.

Despite parents’ general preference to group sports over individual sports, more YP in this study preferred individual sports. This reaffirms the assertion that YP with ASD prefer staying alone and avoiding social situations [10]. Parents reported that when the sport was chosen by the YP, there were improved attendance rates, mirroring previous research [11].

Only one YP was reported to carry out adequate weekly PA in line with WHO (2010) recommendations. This was not surprising given the increasing sedentary lifestyle in YP living in European countries, partly due to their free time being spent surfing the Internet and using technological devices [19]. This was also highlighted through this study’s thematic analysis; YP with ASD who did less PA were reported to engage in more hours of screen time.

Most parents in this study reported a lack of sports opportunities and ASD-friendly facilities for YP with ASD in Malta. Despite the existence of several Maltese agencies, such as Sport Malta (Government of Malta), which compile a directory of sport organisations and facilities [20], parents in this study argued that these facilities are not adequate for YP with ASD. The lack of well-adapted facilities might be due to the lack of public awareness on ASD [21]; as also highlighted in this study’s thematic analysis. Parents reported that sports facilities are located “too far away” or else they “do not find the time” for drop offs and picking up their children. It is also especially difficult for people who rely on public transportation, as YP with ASD might exhibit challenging behaviour when they spend a long time in a motor vehicle [22]. Therefore, sport therapy services should be distributed evenly across the island.

Parents in this study reported that PA has clear benefits as it helps YP with ASD to vent out their anxiety. This might be explained by the notion that PA can serve as a positive coping strategy as an alternative means to repetitive and self-stimulatory behaviour in YP with severe ASD symptoms [23]. This might also be explained by greater parental efforts to find alternatives to more traditional methods to help with ASD symptoms [24]. In fact, all parents in this study showed interest and motivation to improve their child’s PA levels through sports. Therefore, this reflects a disproportionation between the national health service supply and the service users’ demands.

Finally, this study showed that parents were all in favour of PA being beneficial in YP with ASD, and also to themselves as caregivers. This, in despite of the burden of care reported by most parents during the interviews. One may postulate that parents of YP with ASD might be in favour of carrying out PA as they understand that PA is especially important for their own mental wellbeing [25].

### Strengths and Limitations

All interviewers were previously unknown to the participants. The participants were reassured that this research will not, in any way, impact on their healthcare provision, minimizing the Hawthorne effect. Observation bias was minimised by involving research assistants (junior doctors) when completing the interviews, inter-rater exercise, and focus group. Furthermore, inductive thematic analysis aided in representing both the salient and the subtle experiences from the interviews. Translation and back-translation made the interview guide available in both Maltese and English, and a readability test was carried out. The parents came alone for the interviews, giving them the chance to express themselves freely with less emotional barriers. This was the first original study of its kind in Malta.

Selection bias could have been introduced since the sample was taken only from the filing records of the national health service, excluding those YP with ASD who attended private clinics. However, this study focused on improving the Maltese national health service, and the chosen records were the most representative set of data for the general population. Another potential limitation involved the lack of a standardised questionnaire or interview guide in the literature. To minimize researcher bias, the interview guide was created based on the research question, which was tested in a focus group, piloted during an inter-rater exercise, discussed with the clinical lead of the Malta child and adolescent national mental health service, and assessed for readability.

## 5. Conclusions

This study reports that only one young person out of 10 YP with ASD carried out PA in line with WHO recommendations. More parents preferred group PA for their children, while more YP with ASD preferred individual PA. Despite this, parents reported benefits with both group and individual PA. Most parents felt ‘misunderstood,’ ‘burnt out’, and reported ‘a lack of ASD-friendly sports facilities’. Long hours of screen time were a major parental concern.

Recommendations for practice include the introduction of strategic planning for service development of ASD-friendly sports facilities and training of these staff. Furthermore, sport mental health therapy services should be introduced within the national health service, supported by a multidisciplinary team of professionals. This must include parental support services to minimise the burden of care. PA programs in schools should be developed in parallel with mental health services for preventive practice. Recommendations for educational systems include the emphasis on sports mental health courses, public psychoeducation to minimise mental health stigma, and the promotion of healthy use of technological devices to decrease the negative impact of screen time on YP. Using this pilot study, directions for future research include: (1) Validity and reliability testing of a semi-structured interview questionnaire for similar future studies, (2) qualitative research on parents of YP with ASD attending clinics outside the national health service, and (3) a randomised controlled trial including YP with ASD to assess for changes in ASD symptomatology between group and individual PA.

## Figures and Tables

**Table 1:** Phase 1; The Selection Process.

**CYPS Case Referrals 2016–2017**		n = 701
ASD +/− Comorbid Mental Disorders		n = 83
ASD		n = 24
Study Sample		n = 10
ASD Severity	Level 1	n = 2
	Level 2	n = 4
	Level 3	n = 4

**Table 2:** Themes Generated from Thematic Analysis.

169 Initial Codes	
21 Initial Themes	
7 final themes	
Group sports, social interaction, and communication Individual sports, self-esteem and venting one’s frustration Lack of public awareness, opportunities, and resources	4. Lack of motivation in sports 5. Physical and mental burden on relatives 6. General improvement in physical and mental health 7. Technology and screen time

**Table 3:** Results Showing Mean Godin Leisure-Time Exercise Questionnaire (GLTEQ) Scores Per Level of Autism Spectrum Disorder (ASD) Severity Group.

Level of ASD Severity	Mean GLTEQ (Parents)	Mean GLTEQ (YP)
Level 1	33.5	29.5
Level 2	30.5	23.75
Level 3	42.75	54.75

**Table 4:** Results Showing the Mean Screen Time with Different Levels of ASD Severity.

Level of ASD Severity	Mean Screen Time (hours/week)
Level 1	48
Level 2	33
Level 3	15.125

## Data Availability

The data presented in this study are available in the manuscript (‘Results’ section) and in ‘Tables’ above.
